# Optimal modularity and memory capacity of neural reservoirs

**DOI:** 10.1162/netn_a_00082

**Published:** 2019-04-01

**Authors:** Nathaniel Rodriguez, Eduardo Izquierdo, Yong-Yeol Ahn

**Affiliations:** School of Informatics, Computing, and Engineering, Indiana University, Bloomington, IN, USA; School of Informatics, Computing, and Engineering, Indiana University, Bloomington, IN, USA; Cognitive Science Program, Indiana University, Bloomington, IN, USA; School of Informatics, Computing, and Engineering, Indiana University, Bloomington, IN, USA; Indiana University Network Science Institute, Bloomington, IN, USA

**Keywords:** Reservoir computing, Neural network, Echo state network, Dynamical systems, Modularity

## Abstract

The neural network is a powerful computing framework that has been exploited by biological evolution and by humans for solving diverse problems. Although the computational capabilities of neural networks are determined by their structure, the current understanding of the relationships between a neural network’s architecture and function is still primitive. Here we reveal that a neural network’s modular architecture plays a vital role in determining the neural dynamics and memory performance of the network of threshold neurons. In particular, we demonstrate that there exists an optimal modularity for memory performance, where a balance between local cohesion and global connectivity is established, allowing optimally modular networks to remember longer. Our results suggest that insights from dynamical analysis of neural networks and information-spreading processes can be leveraged to better design neural networks and may shed light on the brain’s modular organization.

## Author Summary

Understanding the inner workings of the human brain is one of the greatest scientific challenges. It will not only advance the science of the human mind, but also help us build more intelligent machines. In doing so, it is crucial to understand how the structural organization of the brain affects functional capabilities. Here we reveal a strong connection between the modularity of a neural network and its performance in memory tasks. Namely, we demonstrate that there is optimal modularity for memory performance. Our results suggest a design principle for artificial recurrent neural networks as well as a hypothesis that may explain not only the existence but also the strength of modularity in the brain.

## INTRODUCTION

Neural networks are the computing engines behind many living organisms. They are also prominent general-purpose frameworks for machine learning and artificial intelligence applications (LeCun, Bengio, & Hinton, 2015). The behavior of a neural network is determined by the dynamics of individual neurons, the topology and strength of individual connections, and large-scale architecture. In both biological and artificial neural networks, neurons integrate input signals and produce a graded or threshold-like response. While individual connections are dynamically trained and adapted to the specific environment, the architecture primes the network for performing specific types of tasks. The architecture of neural networks varies from organism to organism and between brain regions and is vital for functionality. The orientation columns of the visual cortex that support low-level visual processing (Hubel & Wiesel, 1972) or the looped structure of hippocampus that consolidates memory (Otmakhova, Duzel, Deutch, & Lisman, 2013) are two examples. In machine learning, feed-forward convolutional architectures have achieved superhuman visual recognition capabilities (Ioffe & Szegedy, 2015; LeCun et al., 2015), while recurrent architectures exhibit impressive natural language processing and control capabilities (Schmidhuber, 2015).

Yet, identifying systematic design principles for neural architecture is still an outstanding question (Legenstein & Maass, 2005; Sussillo & Barak, 2013). Here, we investigate the role of modular architectures on memory capacity of neural networks, where we define modules (communities) as groups of nodes that have stronger internal versus external connectivity (Girvan & Newman, 2002).

We focus on modularity primarily because of the prevalence of modular architectures in the brain. Modularity can be observed across all scales in the brain and is considered a key organizing principle for functional division of brain regions (Bullmore & Sporns, 2009) and brain dynamics (Kaiser & Hilgetag, 2010; Moretti & Muñoz, 2013; Müller-Linow, Hilgetag, & Hütt, 2008; Villegas, Moretti, & Muñoz, 2015; Wang, Hilgetag, & Zhou, 2011), and is also considered as a plausible mechanism for working memory through ensemble-based coding schemes (Boerlin & Denève, 2011), bistability (Constantinidis & Klingberg, 2016; Cossart, Aronov, & Yuste, 2003; Klinshov, Teramae, Nekorkin, & Fukai, 2014), gating (Gisiger & Boukadoum, 2011), and through metastable states that retain information (Johnson, Marro, & Torres, 2013).

Here we study the role of modularity based on the theories of information diffusion, which can inform how structural properties affect spreading processes on a network (Mišić et al., 2015). Spreading processes can include diseases, social fads, memes, random walks, or the spiking events transmitted by biological neurons (Boccaletti, Latora, Moreno, Chavez, & Hwang, 2006; Newman, 2003; Pastor-Satorras, Castellano, Van Mieghem, & Vespignani, 2015), and they are studied in the context of large-scale network properties like small-worldness, scale-freeness, core periphery structure, and community structure (modularity; Boccaletti et al., 2006; Newman, 2003; Strogatz, 2001).

Communities’ main role in information spreading is restricting information flow (Chung, Baek, Kim, Ha, & Jeong, 2014; Onnela et al., 2007). However, recent work showed that communities may play a more nuanced role in *complex contagions*, which require reinforcement from multiple local adoptions. It turns out that under certain conditions community structure can facilitate spread of complex contagions, mainly by enhancing initial local spreading. As a result, there is an optimal modularity at which both local and global spreading can occur (Nematzadeh, Ferrara, Flammini, & Ahn, 2014).

In the context of neural dynamics, this result suggests that communities could offer a way to balance and arbitrate local and global communication and computation. We hypothesize that an ideal computing capacity emerges near the intersection between local cohesion and global connectivity, analogous to the optimal modularity for information diffusion.

We test whether this can be true in reservoir computers. Reservoir computers are biologically plausible models for brain computation (Enel, Procyk, Quilodran, & Dominey, 2016; Soriano, Brunner, Escalona-Moran, Mirasso, & Fischer, 2015; Yamazaki & Tanaka, 2007) as well as a successful machine learning paradigm (Lukoševičius & Jaeger, 2009). They have emerged as an alternative to the traditional recurrent neural network (RNN) paradigm (Jaeger & Hass, 2004; Maass, Natschlager, & Markram, 2002).

Instead of training all the connection parameters as in RNNs, reservoir computers train only a small number of readout parameters. Reservoir computers use the implicit computational capacities of a neural reservoir—a network of model neurons. Compared with other frameworks that require training numerous parameters, this paradigm allows for larger networks and better parameter scaling. Reservoir computers have been successful in a range of tasks including time series prediction, natural language processing, and pattern generation, and have also been used as biologically plausible models for neural computation (Deng, Mao, & Chen, 2016; Enel et al., 2016; Holzmann & Hauser, 2010; Jaeger, 2012; Jalalvand, De Neve, Van de Walle, & Martens, 2016; Rössert, Dean, & Porrill, 2015; Soriano et al., 2015; Souahlia, Belatreche, Benyettou, & Curran, 2016; Triefenbach, Jalalvand, Schrauwen, & Martens, 2010; Yamazaki & Tanaka, 2007).

Reservoir computers operate by taking an input signal(s) into a high-dimensional reservoir state space where signals are mixed. We use echo state networks (ESN)—a popular implementation of reservoir computing—where the reservoir is a collection of randomly connected neurons and the inputs are continuous or binary signals that are injected into a random subset of those neurons through randomly weighted connections. The reservoir’s output is read via a layer of read-out neurons that receive connections from all neurons in the reservoir. They have no input back into the reservoir and they act as the system’s output on tasks.

**Figure 1.**
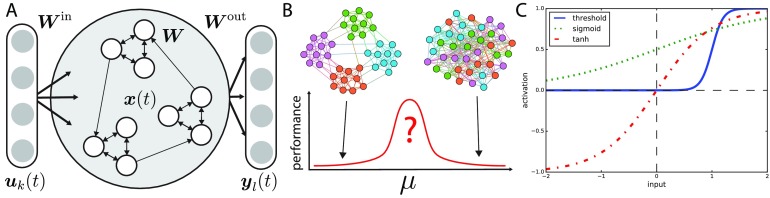
(A) A modular echo state network (ESN). At each time step a *k*-dimensional input signal ***u***_*k*_(*t*) is introduced with randomly weighted input weights ***W***^in^. The reservoir’s state *x*(*t*) evolves through a randomly generated constant weight matrix ***W***. The output weights ***W***^out^ are trained based on the tasks. (B) *μ* is the fraction of bridges that connect communities within the reservoir. At low *μ* community structure is pronounced, while communities vanish at high *μ* (≈ 0.5). We hypothesize that performance increases when a balance between the local cohesion of communities and the global connectivity of bridges is met. (C) A visual comparison of activation functions. Our activation function (solid blue) has threshold-like behavior where small inputs invoke no response up to a threshold, after which the neuron becomes excited. This type of activity mimics the kind expressed in many biological neural networks.

The reservoir weights and input weights are generally drawn from a given probability distribution and remain unchanged, while the readout weights that connect the reservoir and readouts are trained (see Figure 1A). Readout neurons can be considered as “tuning knobs” into the desired set of nonlinear computations that are being performed within the reservoir. Therefore, the ability of a reservoir computer to learn a particular behavior depends on the richness of the dynamical repertoire of the reservoir (Lukoševičius & Jaeger, 2009; Pascanu & Jaeger, 2011).

Many attempts have been made to calibrate reservoirs for particular tasks. In echo state networks this usually entails the adjustment of the spectral radius (largest eigenvalue of the reservoir weight matrix), the input and reservoir weight scales, and reservoir size (Farkas, Bosak, & Gergel, 2016; Jaeger, 2002; Pascanu & Jaeger, 2011; Rodan & Tio, 2011). In memory tasks, performance peaks sharply around a critical point for the spectral radius, whereby the neural network resides within a dynamical regime with long transients and “echos” of previous inputs reverberating through the states of the neurons preserving past information (Pascanu & Jaeger, 2011; Verstraeten, Schrauwen, D’Haene, & Stroobandt, 2007). Weight distribution has also been found to play an important role in performance (Ju, Xu, Chong, & VanDongen, 2013), and the effects of reservoir topology have been studied using small-world (Deng & Zhang, 2007), scale-free (Deng & Zhang, 2007), columnar (Ju et al., 2013; Li, Zhong, Xue, & Zhang, 2015; Maass et al., 2002; Verstraeten et al., 2007), Kronecker graphs (Leskovec, Chakrabarti, Kleinberg, Faloutsos, & Ghahramani, 2010; Rad, Jalili, & Hasler, 2008), and ensembles with lateral inhibition (Xue, Yang, & Haykin, 2007), each showing improvements in performance over simple random graphs.

Echo state networks provide a compelling substrate for investigating the relationship between community structure, information diffusion, and memory. They can be biologically realistic and are simple to train; the separation between the reservoir and the trained readouts means that the training process does not interfere in the structure of the reservoir itself (see the Supporting Information, Table S1; Rodriguez, Izquierdo, & Ahn, 2019).

Here, we take a principled approach based on the theory of network structure and information diffusion to test a hypothesis that the best memory performance emerges when a neural reservoir is at the optimal modularity for information diffusion, where local and global communication can be easily balanced (see the Supporting Information, Figure S1; Rodriguez et al., 2019). We implement neural reservoirs with different levels of community structure (see Figure 1A) by fixing the total number of links and communities while adjusting a mixing parameter *μ* that controls the fraction of links between communities. Control of this parameter lets us explore how community structure plays a role in performance on two memory tasks (see Figure 1B). Three simulations are performed. The first tests for the presence of the optimal modularity phenomena in the ESNs. The second uses the same ESNs to perform a memory capacity task to determine the relationship between the optimal modularity phenomena and task performance. Lastly, we investigate the relationship between community structure and the capacity of the ESN to recall unique patterns in a memorization task.

For the tasks we use a threshold-like activation function (see Figure 1C), which is a more biologically plausible alternative to the tanh or linear neurons often used in artificial neural networks. The key distinction between the threshold-like activation function and tanh activation functions is that threshold-like functions only excite postsynaptic neurons if enough presynaptic neurons activate in unison. On the other hand, postsynaptic tanh neurons will always activate in proportion to presynaptic neurons, no matter how weak those activations are.

## RESULTS

### Optimal Modularity in Reservoir Dynamics

**Figure 2.**
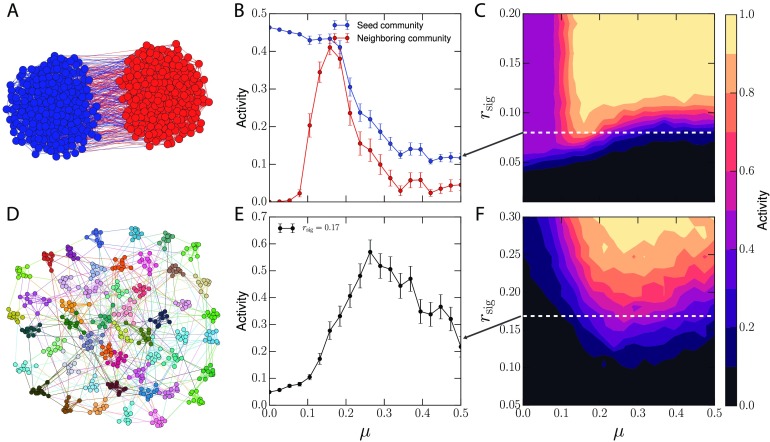
(A) A two-community network of threshold-like neurons receives input into the seed community (blue). (B) An optimal region with maximum activation emerges. (C) Phase diagram for the two-community case. Communities behave similar to gating functions, which can be turned on and transmit information once the input surpasses a threshold. (D) Reservoirs with many communities and randomly injected input also exhibit optimal modularity. (E) The activity level of the network is shown. At low *μ* no single community receives enough signal to be activated, while at high *μ* internal cohesion is too weak to recruit other nodes. In between, the signal can be consolidated effectively, activating larger portions of the network. (F) The full phase-diagram showing the total fractional activity of the network. Error bars represent the standard error of the mean.

We first test whether the optimal modularity phenomenon found in the linear threshold model can be generalized to neural reservoirs by running two simulations. Nodes governed by the linear threshold model remain active once turned on, and are not good units for computing. Instead we use a step-like activation function (see Figure 1C). First, we assume a simple two-community configuration as in the original study (Nematzadeh et al., 2014; see Figure 2A), where the fraction of bridges *μ* controls the strength of community structure in the network. When *μ* = 0, the communities are maximally strong and disconnected, and when *μ* ≈ 0.5 the community structure vanishes. The average degree and the total number of edges remain constant as *μ* is varied. An input signal is injected into a random fraction of the neurons (*r*_sig_) in a seed community and the activity response of each community is measured. The results confirm the generalizability of the optimal modularity phenomenon for neural networks.

At low *μ*, strong local cohesion activates the seed community, while the neighboring community remains inactive as there are too few bridges (see Figure 2B). At high *μ* there are enough bridges to transmit information globally but not enough internal connections to foster local spreading, resulting in a weak response. An optimal region emerges where local cohesion and global connectivity are balanced, maximizing the response of the whole network, as was demonstrated in Nematzadeh et al. (2014) for linear threshold models. The fraction of neurons that receive input (*r*_sig_) modulates the behavior of the communities. The phase diagram in Figure 2C shows how the system can switch from being inactive at low *r*_sig_, to a single active community, to full network activation as the fraction of activated neurons increases. The sharpness of this transition means the community behaves like a threshold-like function as well. Though we control *r*_sig_ as a static parameter in this model, it can represent the fraction of active neural pathways between communities, which may vary over time. Communities could switch between these inactive and active states in response to stimuli based on their activation threshold, allowing them to behave as information gates.

Our second study uses a more general setting, a reservoir with many communities similar to ones that might be used in an ESN or observed in the brain (see Figure 2D). The previous study examined input into only a single community; here we extend that to many communities. In Figure 2E we record the response of a 50-community network that receives a signal that is randomly distributed across the whole network. The result shows that even when there is no designated seed community, similar optimal modularity behavior arises. At low *μ* the input signal cannot be reinforced because of the lack of bridges, and is unable to excite even the highly cohesive communities. At high *μ* the many global bridges help to consolidate the signal, but there is not enough local cohesion to continue to facilitate a strong response. In the optimal region there is a balance between the amplifying effect of the communities and the global communication of the bridges that enables the network to take a subthreshold, globally distributed signal and spread it throughout the network. In linear and tanh reservoirs, no such relationship is found (see the Supporting Information, Figure S2 and Figure S3; Rodriguez et al., 2019); instead communities behave in a more intuitive fashion, restricting information flow.

### Optimal Modularity in a Memory Capacity Task

**Figure 3.**
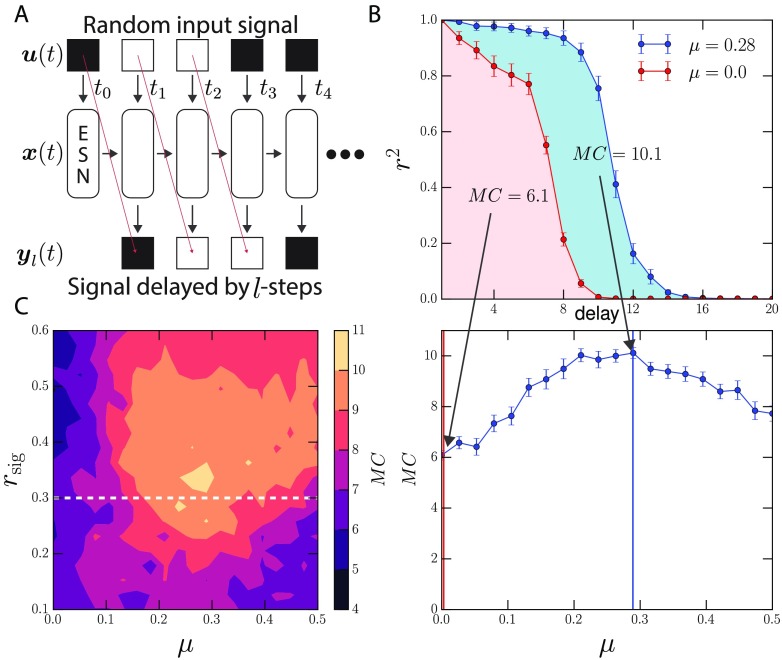
(A) A memory capacity task for measuring the memory duration of ESNs. Readout nodes are trained to reproduce a delayed input sequence. The delay varies from 1 to *l*, where *l* is the number of readouts. (B) Top: The performance is defined by the coefficient of determination (*r*^2^) between the input signal and the output of the node. If the *r*^2^ is 1.0, then the readout perfectly reproduces the inputs. *MC* denotes the overall performance of the ESN on the task. It represents the area under the curve of the *r*^2^ versus delay plot (see shaded regions). (B) Bottom: The average performance over many reservoirs is shown as a function of *μ* where performance is maximal at intermediate levels of modularity. It is taken as a slice through (C) the complete contour-diagram for the task. Error bars represent the standard error of the mean.

We test whether optimal modularity provides a benefit to the ESN’s memory performance by a common memory benchmark task developed by Jaeger (2002; see Figure 3A). The task involves feeding a stream of random inputs into the reservoir and training readout neurons to replay the stream at various time lags. The coefficient of determination between the binomially distributed input signal and a delayed output signal for each delay parameter is used to quantify the performance of the ESN. The memory capacity (MC) of the network is the sum of these performances over all time lags as shown by the shaded region in Figure 3B.

Reservoirs with strong community structure (low *μ*) exhibit the poorest performance; the reservoirs are ensembles of effectively disconnected reservoirs, with little to no intercommunity communication. Performance improves substantially with *μ* as the fraction of global bridges grows, facilitating intercommunity communication. A turnover point is reached beyond which replacing connections with bridges compromises local cohesion. After a certain point, larger *μ* leads to performance loss. The region of elevated performance corresponds to the same region of optimal modularity on a reservoir with the same properties and inputs as those used in the task (see the Supporting Information, Figure S4; Rodriguez et al., 2019).

We also examine the impact of input signal strength. In Figure 3C we show that this optimal region of performance holds over a wide range of *r*_sig_, and that there is a narrow band near *r*_sig_ ≈ 0.3 where the highest performance is achieved around *μ* ≈ 0.2. As expected, we also see a region of optimal *r*_sig_ for reservoirs, because either under- or overstimulation is disadvantageous. Yet, the added benefit of community structure is due to more than just the amplification of the signal. If communities were only amplifying the input signal, then increasing *r*_sig_ in random graphs should give the same performance as that found in the optimal region, but this is not the case. Figure 3C shows that random graphs are unable to meet the performance gains provided near optimal *μ* regardless of *r*_sig_. Additionally, this optimal region remains even if we control for changes in the spectral radius of the reservoir’s adjacency matrix, which is known to play an important role in ESN memory capacity for linear and tanh systems (Farkas et al., 2016; Jaeger, 2002; Verstraeten et al., 2007; see the Supporting Information, Figures S5–S7; Rodriguez et al., 2019). In such systems modularity reduces memory capacity, as communities create an information bottleneck (see the Supporting Information, Figures S8–S9; Rodriguez et al., 2019). However, weight scale still plays a larger role in determining the level of performance for ESNs in our simulations (see the Supporting Information, Figure S5; Rodriguez et al., 2019). There is also a performance difference between the increasingly nonlinear activation functions, with linear performing best, and tanh and sigmoid performing worse, illustrating a previously established trade-off between memory and nonlinearity (Dambre, Verstraeten, Schrauwen, & Massar, 2012; Verstraeten, Dambre, Dutoit, & Schrauwen, 2010; Verstraeten et al., 2007). Lastly, ESN performance has been attributed to reservoir sparsity in the past (Jaeger & Hass, 2004; Lukoševičius, 2012), however as node degree, average node strength, and total number of edges remain constant as *μ* changes such effects are controlled for.

### Optimal Modularity in a Recall Task

**Figure 4.**
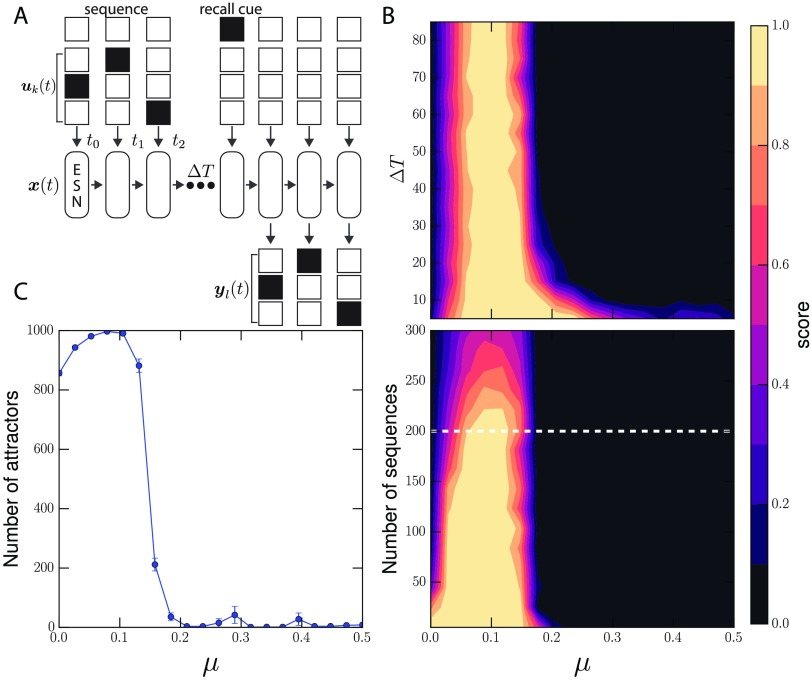
(A) A recall task for testing the amount of patterns that the ESN can learn. For this task, a randomly generated sequence of binary inputs across several dimensions are fed into the reservoir. After Δ*T* time steps, when it receives a cue, it must reproduce the original input sequence. The ESN is trained on each sequence. Performance on the recall task is determined by the fraction of perfect recalls from the learned sequences. A score of 1.0 means that all learned sequences were correctly recalled. (B) Top: Performance is measured against Δ*T*, displaying the maximal performance at *μ* ≈ 0.1. (B) Bottom: The number of sequences that the ESNs can remember for long periods (Δ*T* = 80) shows a similar optimal region. (C) The best performing, optimally modular networks have many more available attractors. Error bars represent the standard error of the mean.

We employ another common memory task that estimates a different feature of memory: the number of unique patterns that can be learned. This requires a rich attractor space that can express and maintain many unique sequences. From here out we consider an attractor to be a basin of state (and input) configurations that lead to the same fixed point in the reservoir state space. In this task, a sequence of randomly generated 0s and 1s are fed to the network as shown in Figure 4A. For the simulation, we use sets of 4 × 5 dimensional binary sequences as input. The readouts should then learn to recall the original sequence after an arbitrarily long delay Δ*T* and the presentation of a recall cue of 1 (for one time step) through a separate input channel.

By varying *μ* we can show how recall performance changes with community structure. Figure 4B, top, shows the average performance measured by the fraction of perfectly recalled sequences, for a set of 200 sequences. Well-performing reservoirs are able to store the sequences in attractors for arbitrarily long times. Similar to the memory capacity task, we see the poorest performance for random networks and networks with low *μ*. There is a sharp spike in performance near *μ* ≈ 0.1. The average performance over the number of sequences (when Δ*T* = 80) show that optimal performance at *μ* starts to drop off after ≈ 230 sequences (Figure 4B, bottom).

We investigate the discrepancy in performance between modular and nonmodular networks by examining the reservoir attractor space. We measure the number of unique available attractors that the reservoirs would be exposed to by initializing the reservoirs at initial conditions associated with the sequences we use. We find a skewed response from the network as shown in Figure 4C where the number of available attractors is maximized when *μ* > 0. Many of these additional attractors between 0.0 < *μ* < 0.2 are limit cycles that result from the interaction between the communities in the reservoir.

The attractor space provides insights about the optimal region. At higher *μ* the whole reservoir behaves as a single system, leaving very few attractors for the network to utilize for information storage. The reservoir has to rely on short-lived transients for storage. With extremely modular structure (*μ* ≈ 0), reservoirs have the most available attractors, but they are not readily discriminated by the linear readouts. Surprisingly, these attractors are more readily teased apart as communities become more interconnected. However, there is a clear trade-off, as too much interconnection folds all the initial conditions into a few large attractor basins.

## DISCUSSION

Biological neural networks are often modeled using neurons with threshold-like behavior, such as integrate-and-fire neurons, the Grossberg-Cohen model, or Hopfield networks. Reservoirs of threshold-like neurons, like those presented here, provide a simple model for investigating the computational capabilities of biological neural networks. By adopting and systematically varying topological characteristics akin to those found in brain networks, such as modularity, and subjecting those networks to tasks, we can gain insight into the functional advantages provided by these architectures.

We have demonstrated that ESNs exhibit optimal modularity in the context of both signal spreading and memory capacity, and they are closely linked to the optimal modularity for information spreading. Through dynamical analysis we found that balancing local and global cohesion enabled modular reservoirs to spread information across the network and consolidate distributed signals, although alternative mechanisms may also be in play, such as cycle properties (Garcia, Lesne, Hilgetag, & Hütt, 2014). We then showed that such optimal regions coincide with the optimal community strength that exhibit the best memory performance. Both the memory capacity and recall task benefited by adopting modular structures over random networks, despite performing in different dynamical regimes (equilibrium versus nonequilibrium).

A key component of our hypothesis is the adoption of a threshold-like (or step-like) activation function for our ESNs, which is a more biologically plausible alternative to the tanh or linear neurons often used in artificial neural networks. The optimal modularity phenomenon emerges only for neural networks of threshold-like neurons and does not exist for neural networks of linear or tanh neurons (i.e., simple contagions) used in traditional ESNs, and so many developed intuitions about ESN dynamics and performance may not readily map to ESNs driven by complex contagions like the ones here. Indeed, the relationship between network topology and performance is known to vary with the activation function, with threshold-like or spiking neurons (common in liquid state machines; Maass et al., 2002) being more heavily dependent on topology (Bertschinger & Natschläger, 2004; Haeusler & Maass, 2007; Schrauwen, Buesing, & Legenstein, 2009). Because the effects of modularity vary depending upon the activation function, a suitable information diffusion analysis should be chosen to explore the impact of network topology for a given type of spreading process. Moreover, because the benefits of modularity are specific to threshold-like neurons, distinct network design principles are needed for biological neural networks and the artificial neural networks used in machine learning. Additionally, as we have seen that the choice of architecture can have a profound impact on the dynamical properties that can emerge from the neural network, there may be value in applying these insights to the architectural design of recurrent neural networks in machine learning, where all weights in the network undergo training but where architecture is usually fixed.

While weight scale remains the most important feature of the system in determining performance, our results suggest significant computational benefits of community structure, and contributes to understanding the role it plays in biological neural networks (Bullmore & Sporns, 2009; Buxhoeveden & Casanova, 2002; Constantinidis & Klingberg, 2016; Hagmann et al., 2008; Hilgetag, Burns, O’Neill, Scannell, & Young, 2000; Meunier, Lambiotte, & Bullmore, 2010; Shimono & Beggs, 2015; Sporns, Chialvo, Kaiser, & Hilgetag, 2004), which are also driven by complex contagions and possess modular topologies. The dynamical principles of information spreading mark trade-offs in the permeability of information on the network that can promote or hinder performance. While this analysis provides us some insight, it remains an open question as to whether our results can be generalized to the context of more realistic biological neural networks where spike-timing-dependent plasticity and neuromodulation play a key role in determining the network’s dynamical and topological characteristics.

In addition to the optimal region and the ability of communities to foster information spreading and improved performance among threshold-like neurons, modularity may play other important roles. For instance, it offers a way to compartmentalize advances and make them robust to noise (e.g., the watchmaker’s parable; Simon, 1997). Modularity also appears to confer advantages to neural networks in changing environments (Kashtan & Alon, 2005), under wiring cost constraints (Clune, Mouret, & Lipson, 2013), when learning new skills (Ellefsen, Mouret, & Clune, 2015), and under random failures (Kaiser & Hilgetag, 2004). These suggest additional avenues for exploring the computational benefits of modular reservoirs and neural networks. And it is still an open question how community structure affects performance on other tasks like signal processing, prediction, or system modeling.

Neural reservoirs have generally been considered “black-boxes,” yet through combining dynamical, informational, and computational studies it maybe possible to build a taxonomy of the functional implications of topological features for both artificial and biological neural networks. Dynamical and performative analysis of neural networks can afford valuable insights into their computational capabilities as we have seen here.

## METHODS

Our ESN architecture with community structure is shown in Figure 1A. The inputs are denoted as ***u***_*k*_(*t*), which is a *k*-dimensional vector. Each dimension of input is connected to a random subset of neurons in the reservoir. ***x***(*t*) is the *N*-dimensional state vector of the reservoir, where *N* is the number of reservoir neurons. ***y***_*l*_(*t*) represents the states of the *l* readout neurons. The *k* inputs are connected by an *N* × *k* matrix ***W***^in^ to the *N* neurons. The network structure of the reservoir is represented by an *N* × *N* weight matrix ***W***, and the output weights are represented by an *N* × *l* matrix ***W***^out^. The reservoirs follow the standard ESN dynamics without feedback or time constants:

$$x(t+1)=f\left(Wx(t)+W^{\text{in}}u(t+1)\right),$$ (1)

$$y(t)=g\left(W^{\text{out}}\left[x(t):u(t)\right]\right).$$ (2)

Here *f* is the reservoir activation function, *g* is the readout activation function, and [*a* : *b*] denotes the concatenation of two vectors. Often *f* is chosen to be a sigmoid-like function such as tanh, while *g* is often taken to be linear (Lukoševičius & Jaeger, 2009). However in our case we use a general sigmoid function:

$$f(z)=\frac{a}{b+e^{-k(z-c)}}-d,$$ (3)

with parameters *a* = 1, *b* = 1, *c* = 1, *k* = 10, and *d* = 0 giving a nonlinear threshold-like activation function, making it step-like in shape and a *complex contagion* like other neuron models (e.g., integrate-and-fire, Hopfield, or Wilson-Cowan models). For the readout neurons, *g* is chosen to be a step function:

$$g(z)=\left\{\begin{array}{ll} 0 & z\leq 0.5,\\ 1 & z>0.5. \end{array}\right.$$ (4)

Linear regression is used to solve for ***W***^out^. ***W***^out^ = ***Y***^tar^***X***^+^ where ***Y***^tar^ is an *l* × *T* matrix of target outputs over a time course *T*, and ***X***^+^ is the pseudoinverse of the history of the reservoir state vector (where ***X*** ∈ ℝ^*N*×*T*^; Lukoševičius & Jaeger, 2009). To generate the reservoirs we use the LFR benchmark model (Lancichinetti, Fortunato, & Radicchi, 2008), which can generate random graphs with a variety of community structures. The LFR benchmark model uses a configuration model to generate random graphs. The configuration model works by imposing a degree sequence to the nodes and randomly wiring the edge “stubs” (Newman, 2010). The LFR model extends this by including community assignment and rewiring steps to constrain the fraction of bridges in the network. Because of its relationship with the configuration model, LFR graphs exhibit low average shortest path length and low average clustering coefficient in contrast to the Wattz-Strogatz models that have low average shortest path length and high clustering. For small graphs like the ones we use for building reservoirs, the average shortest path length increases monotonically with decreasing *μ*. This is due to the sparseness of directed links between communities. As *μ* approaches 0 the communities become disconnected. In our case we vary the fraction of bridges (*μ*) in the network while holding the degree distribution and total number of edges the same, controlling for the density of connections in the network. Weights for the network are drawn separately from a uniform distribution and described in following sections. Code for all the simulations and tasks is available online (Rodriguez, 2018).

### Reservoir Dynamics

We used reservoirs with *N* = 500 nodes, with every node having a degree of 6. Reservoir states were initialized with a zero vector, ***x***(0) = {0, …, 0}. The first experiment uses a two-community cluster of 250 nodes each, matching the scenario from Nematzadeh et al. (2014). Input was injected into *r*_sig_ fraction of neurons into the seed community. The input signal lasted for the duration of the task until the system reached equilibrium at time *t*_*e*_. The final activation values of the neurons were summed within each community and used to calculate the fractional activation of the network for each community shown in Figure 2B, where the mean over 48 reservoir realizations is shown. All activations were summed and divided by the size of the network to give the total fractional activation 1/*N*$\sum_{i=1}^{N}$ *x*_*i*_(*t*_*e*_) as shown in Figure 2C.

In the following experiment, a reservoir of the same size but with 50 communities with 10 nodes each was used. This time, however, the input signal was not limited to a single community but applied randomly to nodes across the network. Again the signal was active for the full duration of the task until the system reached equilibrium when the final activation values of the neurons were summed within each community. Figure 2E shows the activation for each community averaged over 48 reservoir realizations, and the total fractional activity in the network is then shown in Figure 2F.

Different measures for information spreading produce similar results. Also, optimal spreading can be observed in the transitory dynamics of the system, such as in networks that receive short input bursts and return to an inactive equilibrium state. Optimality for step-like activations has been shown to emerge regardless of community or network size using message-passing approximations (Nematzadeh, Rodriguez, Flammini, & Ahn, 2018). For many-community cases with distributed input, optimality existence in infinite networks depends upon community variation (e.g., size, edge density, number of inputs).

### Memory Capacity task

The memory capacity task involves the input of a random sequence of numbers that the readout neurons are then trained on at various lags (see Figure 3). There is just one input dimension and values of 0 and 1 are input into a fraction of the reservoir’s neurons *r*_sig_. For each time lag there is a set of readout neurons that are trained independently to remember the input at the given time lag. The readout neurons that maximize the coefficient of determination (or the square of the correlation coefficient) between the input signal and lagged output are used as the *k*th delayed short-term memory capacity of the network MC_*k*_. The MC of the ESN becomes the sum over all delays:

$$\text{MC}=\overset{\infty }{\underset{k=1}{\sum }}{\text{MC}}_{k}=\overset{\infty }{\underset{k=1}{\sum }}\frac{\text{cov}{\left(u(t-k),y_{k}(t)\right)}^{2}}{\text{var}(u(t))\text{var}(y_{k}(t))}.$$ (5)

We operationalize this sum as the memory capacity of the network. Unlike Jaeger’s task, we input a binomial distribution of 1s and 0s rather than continuous values (see Figure 3A). We try to keep the network small enough and sparse enough to reduce computational load, while still being large enough to solve the task. A reservoir of *N* = 500 nodes and 50 communities of size 10 were used. Every node has a degree of 6. The degree was chosen to be sparse enough to help reduce computing time, while high enough to support a wide range of modularities, which are partly constrained by degree. Reservoir parameters were not fitted to the task, rather a grid search was executed to find parameter sets that performed well, as the focus of the experiment is not to break records on memory performance, but rather to see how it changes with modularity. Among the parameters adjusted were the upper and lower bounds of the weight distribution and the weight scale (*W*_*s*_), which adjusts the strengths of all the reservoir weights by a scalar value. Performance over the full range of *μ* values was evaluated at each point on the grid. Well-performing reservoirs were found with weights between −0.2 and 1 and with a weight scale parameter of *W*_*s*_ = 1.13. The same was done for the input weight matrix, where ***W***^in^ also varies from −0.2 to 1 with an input gain of *W*_*I*_ = 1.0. Many viable parameters existed throughout the space that exhibit optimality. This is partly due to parameter coupling, where changing multiple parameters results in the same dynamics.

Each reservoir’s readouts were trained over a 1,500-step sequence following the first 500 steps that are removed to allow initial transients to die out. Once trained, a new validation sequence of the same length is used to evaluate the performance of the ESN. Results averaged over 64 reservoir samples are shown in Figures 3B and 3C. We also show the contour over *r*_sig_, which is an important parameter in determining the performance of the reservoir. Performance peaks between *r*_sig_ = 0.3 and *r*_sig_ = 0.4 at a *μ* ≈ 0.25.

### Recall Task

The recall task is a simplified version of the memory task developed by Jaeger (Jaeger, 2012). A pattern of 0s and 1s is input into the network, which must recall that pattern after a distractor period. The ESN is trained on the whole set of unique sequences and the performance of the ESN is determined from its final output during the recall period, which occurs after the distractor period. We do this to estimate the total number of sequences that an ESN can remember. So unlike the memory capacity task that estimates memory duration given an arbitrary input sequence, the recall task quantifies the number of distinct signals an ESN can differentiate. This involves training an ESN on a set of sequences and then having it recall the sequences perfectly after a time delay Δ*T*. The input is a random 4 × 5 binary set of 0s and 1s. At a single time step just one of the four input dimensions are active. This is in order to maintain the same level of external excitation per time step, as we are not testing the network’s dynamic range. The reservoir is initialized to a zero vector and provided with a random sequence. Following the delay period, a binary cue with value 1.0 is presented via a fifth input dimension. After this cue, the reservoir’s readout neurons must reproduce the input sequence. The readout weights are trained on this sequence set. Figures 4B shows the average performance over 48 reservoir samples. Many networks around the optimal *μ* value can retain the information for arbitrarily long times, as the task involves storing the information in a unique attractor. Figures 4B shows the average performance when Δ*T* = 80 as we vary the number of sequences. In Figures 4C we determine the average number of available attractors given inputs drawn from the full set of 4 × 5 binary sequences where only one dimension of the input is active at a given time. For each of the 4 × 5 binary sequences, the system was run until it reached the cue time, where a decision would be made by the readout layer. At this point converged trajectories would result in a failure to differentiate patterns. Two converged trajectories are determined to fall into the same attractor if the Euclidean distance between the system’s states are smaller than a value *ϵ* = 0.1. The number of attractor states is the number of these unique groupings and was robust to changes in *ϵ*. Parameters for the reservoir are chosen via a grid search, as before, to find reasonable performance from which to start our analysis. Here reservoirs of size *N* = 1,000 with node degree 7 and community size 10 are used. A larger reservoir was necessary in order to attain high performance on the task. Similarly, the weight distribution parameters are included in the search and reasonable performing reservoirs were found with weights drawn between −0.1 and 1.0 with *W*_*s*_ = 1.0, *r*_sig_ = 0.3, an input gain of *W*_*I*_ = 2.0, and uniform input weights of 1.0.

## ACKNOWLEDGMENTS

We would like to thank John Beggs, Alessandro Flamini, Azadeh Nematzadeh, Pau Vilimelis Aceituno, Naoki Masuda, and Mikail Rubinov for helpful discussions and valuable feedback. This research was supported in part by Lilly Endowment, Inc., through its support for the Indiana University Pervasive Technology Institute, and in part by the Indiana METACyt Initiative. The Indiana METACyt Initiative at IU was also supported in part by Lilly Endowment, Inc. The Indiana University HPC infrastructure (Big Red II) helped make this research possible.

## AUTHOR CONTRIBUTIONS

Nathaniel Rodriguez: Conceptualization; Formal analysis; Methodology; Software; Validation; Visualization; Writing - Original Draft; Writing - Review & Editing. Eduardo Izquierdo: Conceptualization; Methodology; Supervision; Writing - Original Draft; Writing - Review & Editing. Yong-Yeol Ahn: Conceptualization; Methodology; Supervision; Writing - Original Draft; Writing - Review & Editing.

## TECHNICAL TERMS

Complex contagions: Contagion where spreading is enabled by reinforcement from other contagions, such as spiking neurons, as opposed to diseases or random walks.
Reservoir: A system that carries out (often nonlinear) computations on some input signal.
Echo state network: A type of reservoir computer that relies on a system of neurons to perform nonlinear computations on an input signal.
Threshold model: A type of complex contagion where spreading occurs only after a set proportion or number of neighbors become active.
Attractor: A region in state space where all states converge upon a single fixed point or cycle.
