# Supplementary material for: Optimal modularity and memory capacity of neural reservoirs
Source: Netw Neurosci. 2019 Apr 1;3(2):551–66. doi: 10.1162/netn_a_00082 (PMC6497001; doi:10.1162/netn_a_00082)
Supplement: Supplementary file 1 [file netn-03-551-s001.pdf]

Rodriguez, N., Izquierdo, E., & Ahn, Y.-Y. (2019). Supporting information for "Optimal modularity and memory capacity of neural reservoirs." *Network Neuroscience*, 3(2), 551–566. https://doi.org/10.1162/netn_a_00082

Technique	Realism	Training	Structural Control	Task Flexibility
ESN	model choice	simple, convex	full	high
RNN	model choice	complex, non-convex	low	low
LSTM	low	complex, non-convex	low	low

TABLE S.1. A break-down of the differences between ESNs and a couple other artificial neural network approaches that can be trained on complex tasks. ESNs provide an ideal environment for testing our hypothesis due to their flexibility. Traditional RNNs and LSTMs (long-short term memory neural networks) require more complex training procedures which alter the synaptic weights between neurons resulting in loss of control over network structure impacting modularity and other network properties. Alternatively, reservoirs in ESNs are static allowing us to readily control the properties of the network that we want to investigate. Additionally, ESN reservoirs can be swapped from task to task without modification due to the separate input and read-out layers, whereas RNNs and LSTMs need entirely new networks for each task. Lastly, ESNs and RNNs can be made more biologically realistic by including higher fidelity models for their components, such as spiking neurons or plastic synapses. We opted against this due to the added complexity that comes with continuous-time spiking neurons which require special encoding/decoding rules for task inputs and outputs.

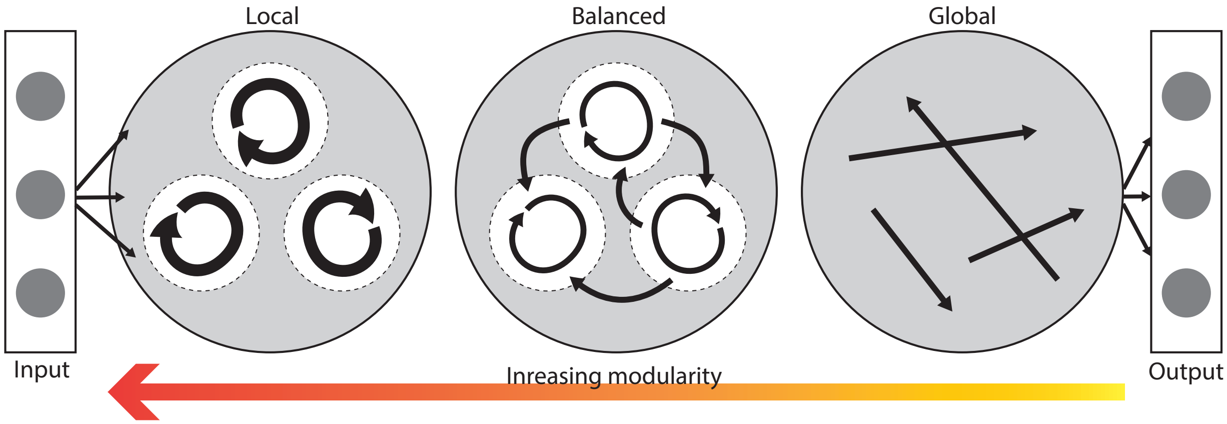

FIG. S.1. The modularity of the reservoir of the ESN can vary from tightly coupled communities to a completely random network. In the “local” domain information travels freely within communities but has difficulty escaping and isn’t shared between communities. Alternatively, in the “global” domain neurons have connections throughout the reservoir and information should flow freely, but due to the lack of local cohesion provided by communities neurons fail to activate and information flow is quenched. The read-out layer of the ESN is connected to all the neurons in the reservoir, however it is only a linear aggregation of the reservoir state. To take advantage of the non-linearity of the reservoir information would need to be maintained and processed within it. We argue that these benefits are best achieved in a balanced region where local cohesion and global connectivity facilitate global information spreading.

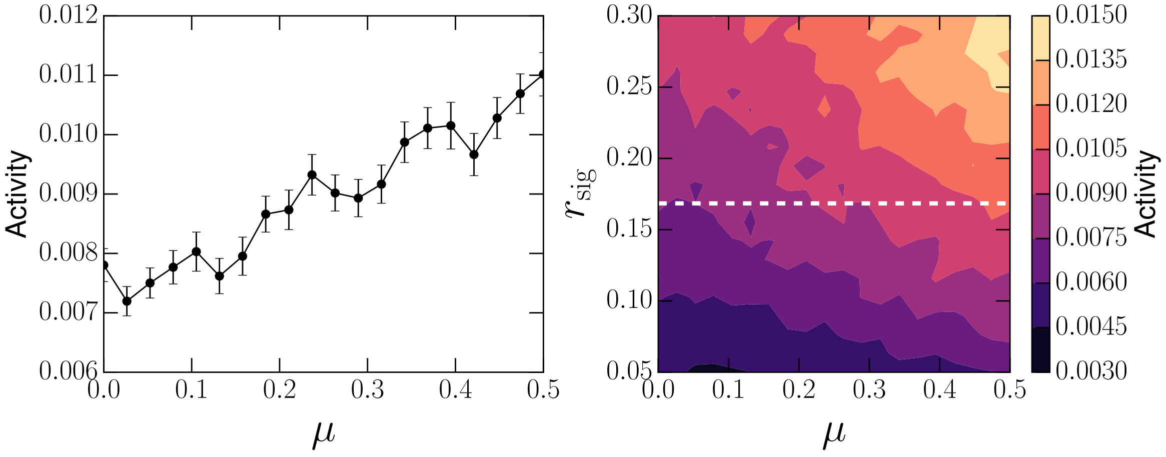

FIG. S.2. The absolute value of the activity response of a linear reservoir. As μ decreases so does the activity. (left) Shows a slice through the phase diagram at the dashed white line. (right) The activity as μ and input fraction vary. It uses graphs with $N = 500$, $k = 6$, a community size of 10. The spectral radius has been shown to have a large impact on linear and tanh reservoir behavior, so we correct for changes in spectral radius as μ changes. The reservoir is kept just below the critical point with $W_s = 0.9$ to prevent numerical explosions.

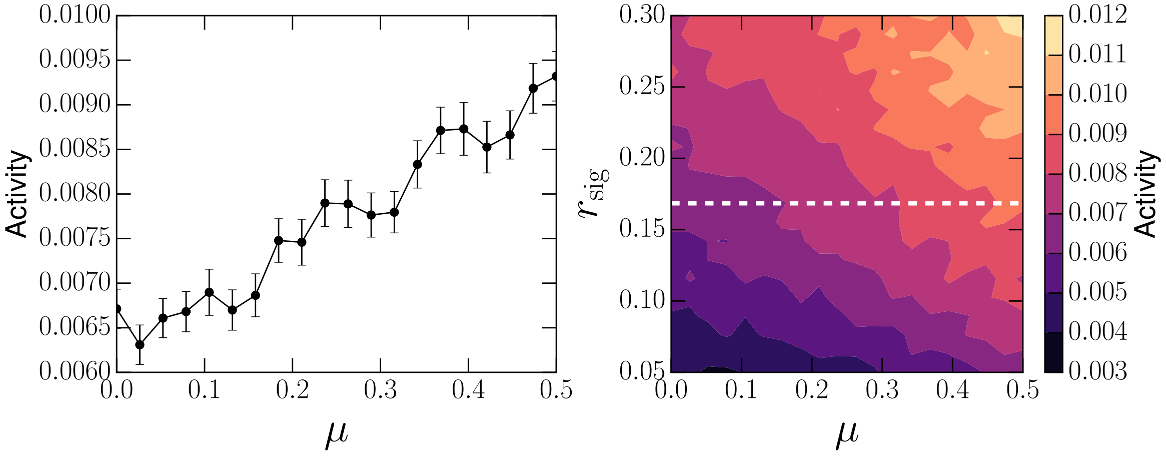

FIG. S.3. The absolute value of the activity response of a tanh reservoir. As μ decreases so does the activity. (left) Shows a slice through the phase diagram at the dashed white line. (right) The activity as μ and input fraction vary. It uses graphs with $N = 500$, $k = 6$, a community size of 10. The spectral radius has been shown to have a large impact on linear and tanh reservoir behavior, so we correct for changes in spectral radius as μ changes. The reservoir is kept at the critical point with $W_s = 1.0$.

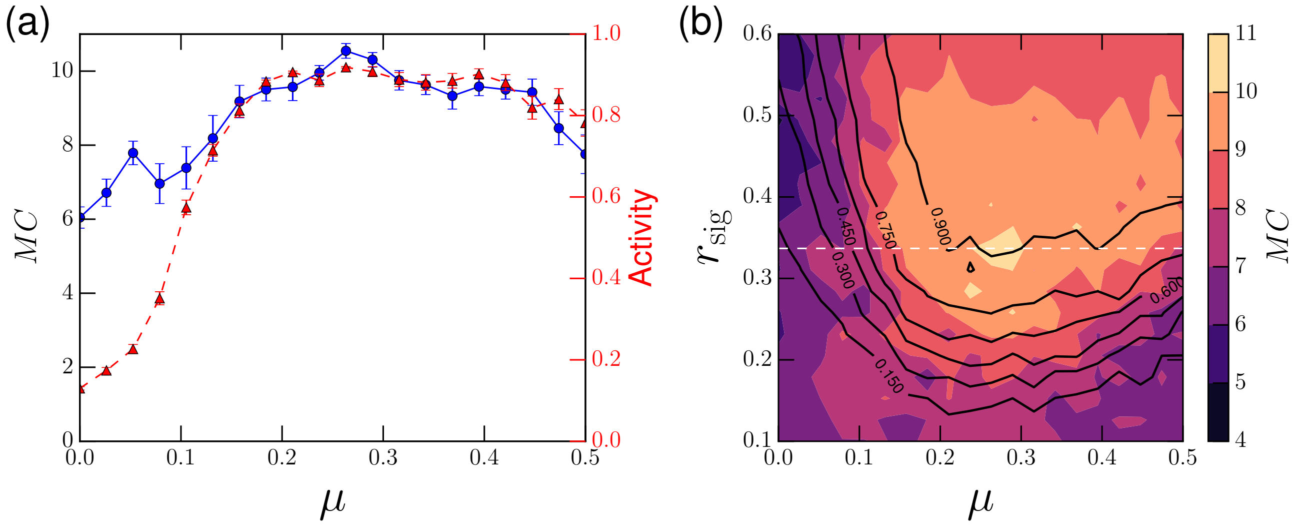

FIG. S.4. To verify that optimal μ for both performance and diffusion coincide, we overlay the contours of the MC task and the information diffusion process. (a) A slice through the contour at $r_{\rm sig} = 0.3$ shows that the normalized activity is maximized when MC is maximized. (b) The solid black lines show the contours for the information diffusion process, closely matching the MC contours for performance on the task. In this information diffusion task an input identical to the one given the MC task is used so that $r_{\rm sig}$ correspond to the same levels of activation the network would receive during the MC task. The total sum of activation is then used, normalized by the duration of the test and the size of the network. Error bars represent the standard error of the mean.

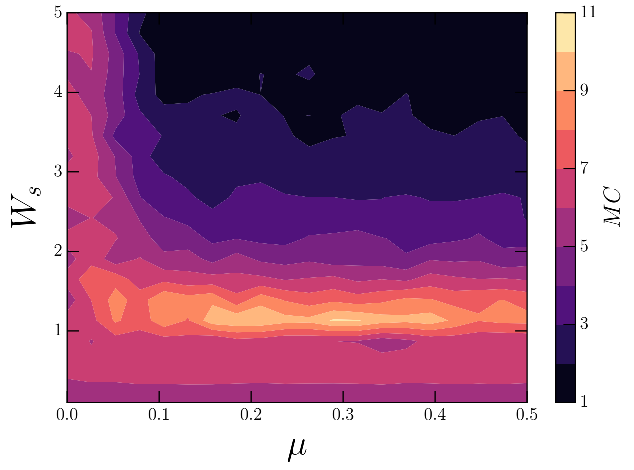

FIG. S.5. A contour of the MC performance on the same network as in the main-text for the MC task while holding $r_{\rm sig} = 0.3$ and varying over the parameter W_s, a constant factor that the weight matrix $\boldsymbol{W}$ is multiplied by. Best performance occurs within a narrow band of weights. Importantly, there are no weights either above or below the optimal μ region where performance reaches the same level, suggesting that controlling for the size of the largest eigenvalue of the adjacency matrix will be insufficient to explain the performance gains afforded by the modularity.

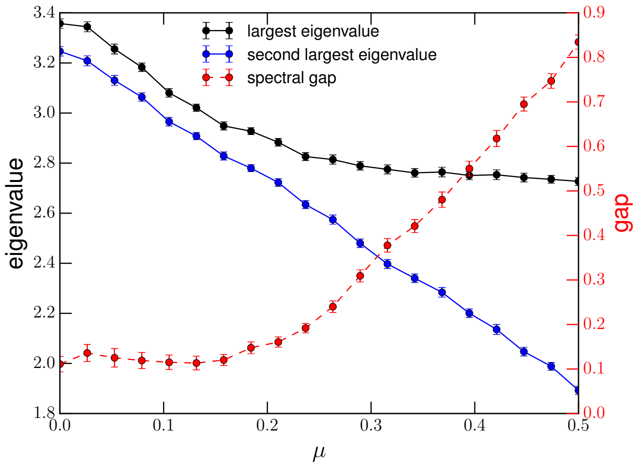

FIG. S.6. The absolute values of the largest and second largest eigenvalues of the adjacency matrix and spectral gap are plotted for the same network used in the MC task. At low μ the gap is small and both the first and second eigenvalues decrease at roughly the same rate as μ increases. However, at roughly $\mu \approx 0.25$ the rate of decline of the largest eigenvalue flattens out and the gap increases as the network approaches a traditional random graph. Error bars represent the standard error of the mean.

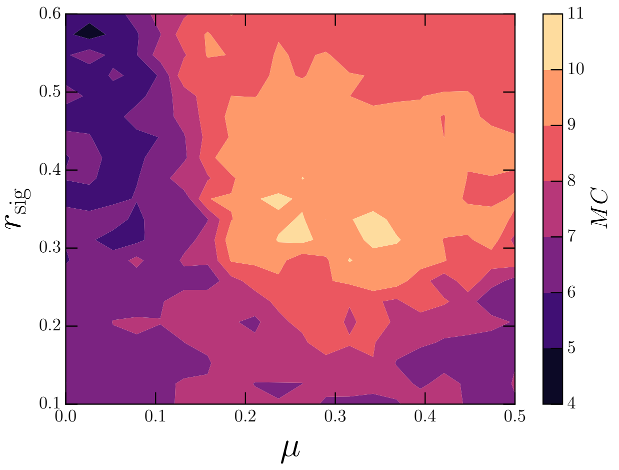

FIG. S.7. We can correct for the changes in the spectral radius as μ is changed by holding the absolute value of the largest eigenvalue constant. Qualitatively we get the same results even while maintaining a constant spectral radius. The inability of the spectral radius to account for the increased memory capacity is likely due to our use of threshold-like activation functions that mimic complex contagions. Traditionally, the strong relationship between spectral radius and memory capacity is linked with traditional tanh activation functions.

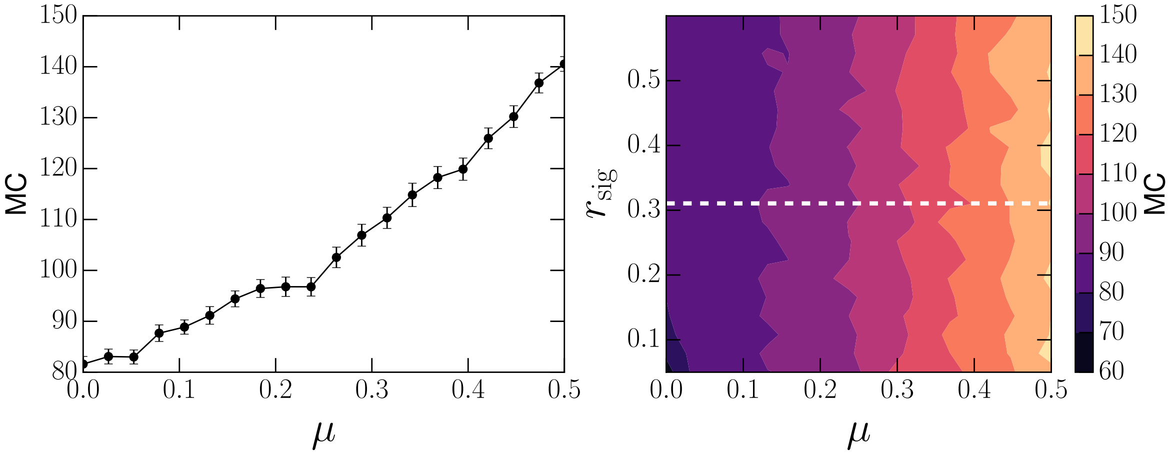

FIG. S.8. The memory capacity (MC) of a linear reservoir. The introduction of community structure degrades memory performance. (left) Shows a slice through the phase diagram at the dashed white line. (right) The memory capacity as μ and input fraction vary. The same graphs and parameters as the activation simulation are used; with $N = 500$, $k = 6$, a community size of 10, a $W_s = 0.9$, and with corrections for the spectral radius.

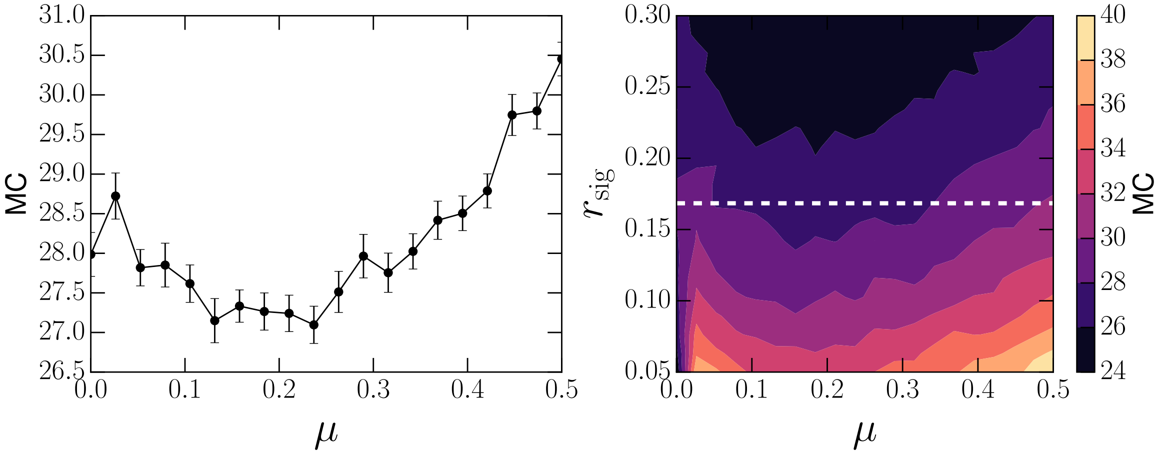

FIG. S.9. The memory capacity (MC) of a tanh reservoir. Similar to the linear reservoir, the introduction of community structure degrades memory performance. As r_{sig} decreases, the memory capacity rises due to the reduced external excitations allowing the reservoir to operate closer to the higher performing linear regime of the tanh activation function. (left) Shows a slice through the phase diagram at the dashed white line. (right) The memory capacity as μ and input fraction vary. The same graphs and parameters as the activation simulation are used; with $N = 500$, $k = 6$, a community size of 10, a $W_s = 1.0$, and with corrections for the spectral radius.
